# An Artificial Intelligence Framework for Detection of Leukemia with Comparison Between Segmented and Non-Segmented Images

**DOI:** 10.3390/s26196290

**Published:** 2026-10-04

**Authors:** Rula Abdallat, Osama Al-Bataineh, Ali Mohammad Alqudah, Ausilah Alfraihat

**Affiliations:** 1 Department of Biomedical Engineering, Faculty of Engineering, The Hashemite University, P.O. Box 330127, Zarqa 13133, Jordan; omabio@hu.edu.jo (O.A.-B.); ausilaha@hu.edu.jo (A.A.); 2 Independent Researcher, Winnipeg, MB R2M 3R1, Canada

**Keywords:** acute lymphoblastic leukemia, convolutional neural network, deep learning, peripheral blood smear, white blood cell classification, class activation maps

## Abstract

Background: Accurate classification of Acute Lymphoblastic Leukemia (ALL) subtypes from peripheral blood smear (PBS) images using deep learning may support automated image-based analysis of this blood malignancy. Methods: We developed a custom lightweight Convolutional Neural Network (CNN) to classify white blood cells into four subtypes: Benign, Early Pre-B ALL, Pre-B ALL, and Pro-B ALL. We assessed performance using accuracy, ROC curves, and explainability techniques, including upsampled feature map activations, occlusion sensitivity, and SHAP-style explanation overlays, comparing original (Non-segmented) and HSV-Segmented images. To mitigate patient-level data leakage without explicit patient identifiers, we further used unsupervised K-means clustering of HSV color histograms to group 3256 images into 89 “proxy-patient” clusters. Consequently, we implemented rigorous subject-level grouped 5-fold cross-validation and Hold-Out protocols alongside standard image-level evaluations. Results: Under standard image-level evaluation, the model achieved 98.8% accuracy on non-segmented images and 91.3% on segmented images. Crucially, this performance advantage was maintained under strict subject-level cross-validation (97.9% vs. 89.7%), confirming the robustness of the findings against data leakage. Explainability visualizations demonstrated that HSV segmentation discarded vital contextual morphological cues, increasing confusion between closely related subtypes. Conclusions: A lightweight CNN can accurately classify ALL subtypes directly from raw PBS images. The evaluated HSV segmentation degraded performance by removing contextual information. This study underscores the necessity of subject-level evaluation in medical imaging and indicates that mandatory preprocessing should be empirically justified rather than assumed beneficial.

## 1. Introduction

Leukemia is a malignancy characterized by an increase and abnormal growth of white blood cells in the bone marrow. This type of cancer is classified into four types: acute lymphocytic leukemia (ALL), chronic lymphocytic leukemia (CLL), acute myelogenous leukemia (AML), and chronic myelogenous leukemia (CML) [1]. The disease can spread to other organs, causing a decline in immune function and the bone marrow’s ability to produce red blood cells and platelets. Thus, it is considered a major health issue with high rates of morbidity and mortality, particularly in cases where diagnosis is late [2]. Registry-based analysis from Jordan indicates a statistically significant decline in leukemia incidence in both adults and children between 2000 and 2018 [3]. Therefore, early diagnosis is crucial to improve survival rates and enable rapid treatment [4]. Traditional diagnostic approaches, such as blood counts, peripheral blood smears (PBS), biopsies, flow cytometry, genetic tests, and imaging, have limitations such as invasiveness, high cost, variability, and time-consuming, labor-intensive processes [1,5,6,7,8].

Recent developments in Artificial Intelligence (AI), such as in machine learning (ML) and deep learning (DL), have improved medical image analysis by allowing automated identification of complex morphological features directly from raw images [9,10,11,12,13]. Therefore, the need for manual feature design and potential human bias is reduced, resulting in better efficiency and accuracy in diagnostic procedures [14]. Deep learning techniques learn and construct layered representations of data. Convolutional Neural Networks (CNNs) are a powerful example in this domain. They are highly effective models for image-based tasks structured to analyze visual data by learning spatial patterns through convolutional operations, consequently allowing them to identify important features within images [15]. This is particularly valuable in medical imaging, where slight variations in cellular morphology can be critical for disease diagnosis [16,17].

The recent literature has studied the ability of deep learning methods, especially CNNs, to classify blood cell images and detect abnormalities connected with various diseases such as sickle cell anemia, malaria, and Thalassemia [18,19,20]. These studies show that AI approaches can provide accurate, automated analysis, highlighting their potential as supportive tools in clinical diagnosis and their ability to improve healthcare outcomes.

Both traditional (ML) and deep learning (DL) methods were used for leukemia image classification. Earlier studies mainly relied on algorithms such as Support Vector Machines (SVMs) and clustering techniques that required manual feature extraction from blood smear images based on morphological characteristics [21,22], including cell shape [23], texture [24], and color [25]. Despite demonstrating the ability to automate leukemia diagnosis, performance was often limited by reliance on manual feature design and variations in image quality and staining conditions [26]. On the other hand, recent work has demonstrated that CNN-based models can achieve very high accuracy in detecting leukemia from microscopic blood smear images, often outperforming traditional methods and hybrid ML approaches [27,28,29]. Table 1 provides representative studies and identifies the dataset used by each. Reported accuracies from different datasets are presented only for context and are not treated as direct benchmarks because image sources, class definitions, preprocessing procedures, and validation protocols differ.

Many AI-based leukemia detection systems rely on preprocessing steps, particularly cell segmentation, to isolate white blood cells before classification [37,38]. Segmentation isolates regions of interest from the surrounding background [39] and is thought to improve classification accuracy by reducing irrelevant information and directing the model’s focus to relevant features [40]. Its effect, however, depends on the segmentation method and the information preserved in the resulting image. Variations in staining, overlapping cells, imaging artifacts, and inaccurate masks can alter the Segmented representation [37,41,42]. Consequently, evaluate the value of segmentation for the particular method, dataset, and classification task rather than assuming it in advance.

Recent advances in deep learning suggest that CNN models can learn features directly from original Non-segmented images [43,44,45]. Although CNN-based approaches have shown promising results in leukemia image analysis [46], fewer studies have examined multiclass classification across the B- ALL [47,48]. In addition, the effect of removing background and contextual information before subtype classification remains insufficiently characterized. This motivates a paired comparison between the original (non-segmented) images and the corresponding HSV-segmented images (Segmented) released with the same public dataset.

This study proposes a custom CNN framework for classifying (PBS) images into four ALL subtypes: Benign (hematogone), Early Pre-B ALL, Pre-B ALL, and Pro-B ALL. We used the Non-segmented and paired Segmented images provided by the dataset creators, generated via HSV color-thresholding, to examine how preprocessing affects model performance and to develop an accurate, efficient automated leukemia diagnosis system. Therefore, the primary scientific novelty of this study does not lie in the proposed CNN architecture itself, but in the rigorous empirical evaluation of contextual information loss in multiclass ALL subtyping. We explicitly quantify how mandatory preprocessing (HSV-based segmentation) destroys the subtle morphological cues required to distinguish closely related maturation stages (e.g., Early Pre-B vs. Pre-B).

## 2. Materials and Methods

**Figure 1:**
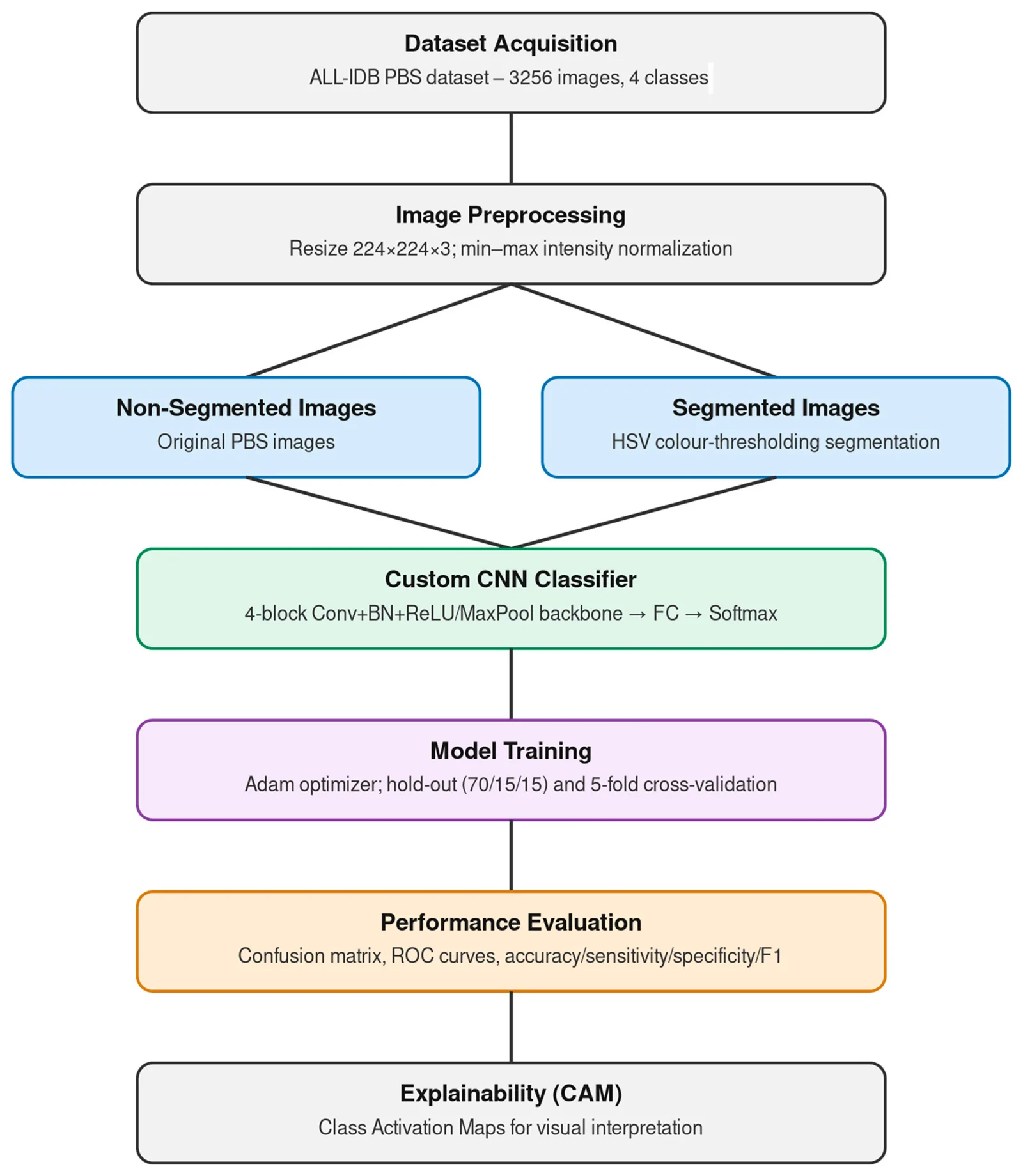
Overview of the proposed methodology, from dataset acquisition and preprocessing (Non-segmented and Segmented branches) through custom CNN classification, dual training/validation protocols, performance evaluation, and upsampled feature map activations.

The methodology includes dataset description, image preprocessing, network architecture design, two training and evaluation protocols (hold-out and 5-fold cross-validation), and an explainability analysis. All experiments were implemented in MATLAB using the Deep Learning Toolbox and executed in a GPU-accelerated environment. An overview of the complete pipeline, from dataset acquisition to explainability analysis, is illustrated in Figure 1.

A. Dataset Description

The image dataset used in this study was originally collected by Aria et al. (2021) [49] and is publicly available on Kaggle (DOI: 10.34740/KAGGLE/DSV/2175623) [49]. In summary, we collected 3256 PBS microscopy images from 89 patients at the bone marrow laboratory of Taleqani Hospital (Tehran, Iran). Skilled laboratory personnel prepared and stained samples under expert hematological supervision. Out of 89 patients, 25 were healthy individuals diagnosed with benign (hematogone) disease, and 64 had a confirmed ALL diagnosis across three malignant subtypes.

The dataset is organized into four classes: (1) Benign non-cancerous hematopoietic lymphoid cells that morphologically resemble ALL blasts, (2) Early Pre-B ALL, the earliest detectable stage of B-lineage ALL, (3) Pre-B ALL, an intermediate subtype, and (4) Pro-B ALL, the most undifferentiated subtype. Table 2 summarizes the distribution of images across these classes, including the number of samples and their corresponding percentages of the total dataset. We used two versions of the dataset: original, unprocessed microscopy images and Segmented images, in which cellular regions of interest were isolated from the background using color-thresholding segmentation in the HSV color space.

Ethics statement: The dataset used in this study is publicly available and fully anonymized. Because this study used publicly available, de-identified data, informed consent and institutional review board approval were not required.

B. Image Preprocessing

Spatial Normalization was applied by uniformly resizing all images to 224 × 224 × 3 pixels (RGB) using bilinear interpolation via MATLAB’s imresize function, applied inline within the datastore’s custom read function. This standardizes spatial dimensions across all samples, satisfying the fixed-size input requirement of the proposed CNN architecture. On the other hand, Pixel Intensity Normalization was applied via a custom normalizeImage function implementing min–max scaling, defined as:

N = (I − I_min)/(I_max − I_min)(1)

where I is the raw pixel intensity, and I_min and I_max are the global minimum and maximum pixel intensities of the input image. This operation maps all pixel values to [0, 1], reducing staining variability across samples. We applied this normalization consistently during both model training and upsampled feature map activations visualization.

For the Segmented variant, cellular regions of interest were isolated using color thresholding in the HSV (Hue, Saturation, Value) color space. Specifically, the Hue channel was thresholded (range: 110–160) to isolate the purple/magenta stains characteristic of leukocyte nuclei, the Saturation channel was thresholded (>100) to exclude achromatic background regions, and the Value channel was thresholded (>80) to remove dark artifacts. After thresholding, morphological opening (disk structuring element, radius 2) removed small noise particles, followed by morphological closing (disk structuring element, radius 3) to fill small holes within the Segmented regions. The resulting binary mask was applied to the original image to produce the Segmented cell [50]. Although more advanced deep learning-based segmentation methods (e.g., U-Net) exist, we selected HSV thresholding as a standard, computationally lightweight, and reproducible baseline preprocessing pipeline. We acknowledge that more sophisticated segmentation approaches may preserve boundary nuances differently and could yield different classification outcomes.

C. Proposed CNN Architecture

**Figure 2:**
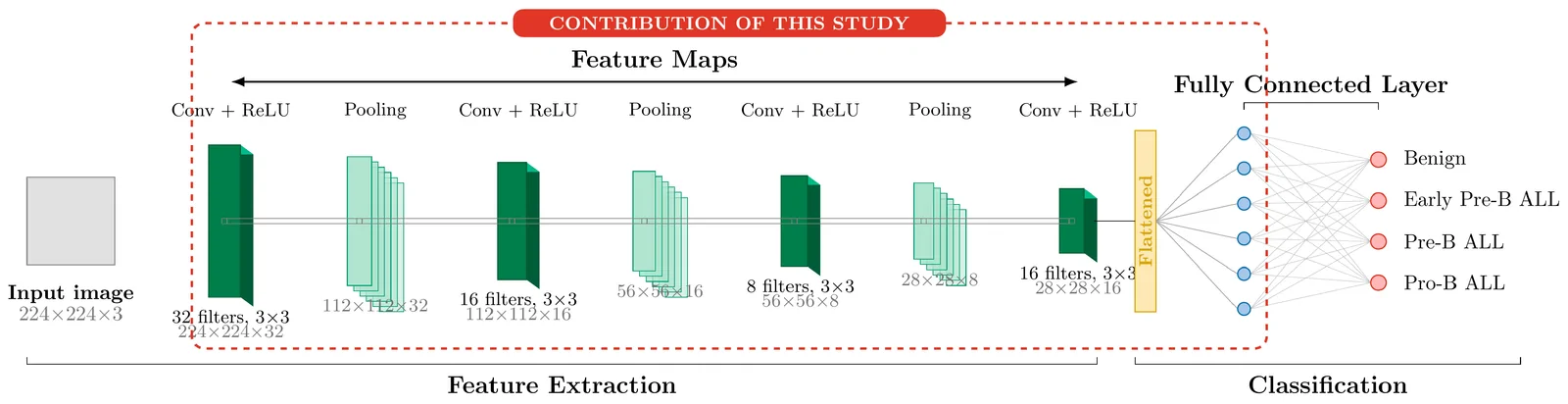
Illustrated architecture of the proposed custom CNN, showing four convolutional stages (32/16/8/16 filters) and three max-pooling stages that extract features from the 224 × 224 × 3 input image, followed by flattening and a fully connected layer producing four ALL subtype predictions (Benign, Early Pre-B, Pre-B, and Pro-B ALL). The dashed outline marks this study’s custom-designed contribution.

We designed a lightweight custom CNN from scratch in MATLAB’s Deep Learning Toolbox. The network accepts 224 × 224 × 3 RGB images. It comprises four sequential convolutional blocks, each consisting of: a 2D Convolution layer (3 × 3 kernel, same padding), a Batch Normalization (BN) layer, and a ReLU activation function. the first three blocks are each followed by a 2 × 2 Max-Pooling layer with stride 2, while the fourth block omits pooling to preserve spatial resolution before flattening. The filter depth follows a funnel-then-expand pattern (32 → 16 → 8 → 16). The network terminates with a Fully Connected (FC) layer of 4 units, a Softmax layer, and a Classification output layer. Batch normalization after each convolutional layer accelerates convergence and reduces internal covariate shift. Three successive max-pooling stages progressively reduce the spatial resolution from 224 × 224 to 28 × 28, capturing hierarchical morphological features of ALL blast nuclei, cytoplasmic boundaries, and chromatin texture across multiple scales. Figure 2 provides a detailed layer-by-layer description of the proposed CNN architecture.

D. Training Configuration

We optimized the network using the Adam (Adaptive Moment Estimation) optimizer, selected for its adaptive learning rate properties and robustness to sparse gradients. Training was conducted with the following hyperparameter settings (Table 3):

As shown, the model was trained for up to 100 epochs with a mini-batch size of 64 and an initial learning rate of 0.001. We resized images to 224 × 224 × 3 (RGB) before training. The dataset was divided into 70% training, 15% validation, and 15% testing. We used 5-fold cross-validation to evaluate model robustness and generalizability. We also shuffled the training data at every epoch and performed validation every 50 iterations.

All training was performed on a CUDA-compatible GPU, with an explicit GPU state reset (gpuDevice/reset) before each training run to prevent memory leakage between folds. Training progress was logged and displayed in real time using MATLAB’s built-in training progress monitor.

E. Experimental Protocols
  E.1. Whole Dataset Protocol

We applied two complementary evaluation strategies independently to both the Non-segmented and Segmented dataset variants to rigorously assess model generalizability. For hold-out validation, the dataset was randomly partitioned into 70% training, 15% validation, and 15% testing subsets using MATLAB’s splitEachLabel function with the ‘randomize’ flag, ensuring class-stratified splits. We evaluated the trained model on the held-out test set and computed confusion matrices for both the training and test sets to assess potential overfitting. We implemented a stratified 5-fold cross-validation (CV) scheme using MATLAB’s cvpartition function with the ‘Stratify’ option enabled and a fixed random seed (rng(42)). This ensures that each fold maintains the same proportion of the four ALL subtypes as the original dataset, eliminating the class-imbalance bias inherent in the previously used systematic index-based partitioning.

  E.2. Subject Grouping via Unsupervised HSV Clustering

Because the public dataset lacks explicit patient-to-image metadata, true patient-disjoint splitting is technically impossible. To mitigate the risk of patient-level data leakage we implemented an unsupervised grouping strategy to approximate patient identity. Images from the same patient typically share identical slide preparation, staining intensity, and microscopy lighting conditions. To capture these batch-specific visual signatures, we extracted 3D HSV (Hue, Saturation, Value) color histograms from each of the 3256 images using 8 bins per channel (512 features). We applied Principal Component Analysis (PCA) to reduce the feature space to the top 50 components. K-means clustering was then performed to partition the dataset into K=89 distinct clusters, corresponding to the known number of patients in the original study. These 89 clusters serve as “proxy patients” for the subsequent evaluation protocols.

  E.3. Subject-Level Grouped Cross-Validation Protocol

To prevent “proxy patient” data leakage between training and testing, we replaced standard image-level random splits with strict Subject-Level partitions. Instead of partitioning individual images, we applied MATLAB’s cvpartition function to the 89 unique cluster IDs. We randomly divided the 89 proxy patients into 5 folds. We strictly assigned all images belonging to a specific cluster to the same fold, ensuring the model was evaluated on entirely unseen “patients” during each testing phase. We applied this protocol independently to both the Non-segmented and Segmented datasets.

F. Performance Evaluation

After completing cross-validation, we aggregated all fold-wise predicted labels and posterior probabilities to produce a global performance assessment. We computed a global confusion matrix using confusionmat and visualized it with plotconfusion. We computed per-class ROC curves and Area Under the Curve (AUC) scores using MATLAB’s perfcurve in a one-vs-rest configuration, generating a 2 × 2 subplot for the four output classes (Table 4).

G. Explainability via upsampled feature map activations, occlusion sensitivity, and SHAP-style explanation overlay visualization.

To provide comprehensive, interpretable visual evidence of the CNN’s decision-making process, we employed a multi-faceted explainability framework. First, Class Activation Maps (CAMs) were generated from the network’s intermediate feature representations to identify globally discriminative regions [51]. Activations were extracted from the final convolutional ReLU layer (relu_4, Layer 16). To address the low-spatial-resolution limitations of standard CAMs (28 × 28), we upsampled the extracted activation maps to the original image resolution (224 × 224) using bicubic interpolation before visualization. We computed per-channel mean activations across spatial dimensions to rank feature-map importance, then selected the top-4 discriminative channels using maxk (scores, 4). The CAM generation pipeline consists of six steps: (1) Normalize the activation map to [0, 1] using min–max scaling; (2) Threshold low activations (values below 0.2 set to zero) to suppress background noise; (3) Colorize using a jet colormap scaled by activation intensity; (4) Blend colorized CAM with the grayscale input (Combined = I_gray/2 + CAM_colored); (5) Re-normalize the combined image to [0, 255] and render as uint8; and (6) Display in a 2 × 3 subplot alongside class labels and confidence scores.

Second, to evaluate local feature importance through perturbation, we employed occlusion sensitivity analysis [52]. This method systematically occludes small rectangular patches of the input image (Mask Size: 15 × 15 pixels) with a stride of 5 pixels. It measures the drop in the predicted class probability at each position. Regions where occlusion causes a significant probability drop are identified as critical for the model’s prediction, providing parameter-driven local interpretability without requiring a separate surrogate model.

Third, to further enhance interpretability, we implemented a SHAP-style explanation overlay [53], while exact SHAP-style explanation overlay value computation requires computationally prohibitive coalitional game theory permutations [54]; this SHAP-style explanation overlay inspired visualization leverages activations from the final convolutional layer (relu_4) to achieve equivalent pixel-level interpretability. We normalized the upsampled activation map to [−1, 1]. To mimic the SHAP-style explanation overlay’s diverging color convention, we mapped positive values (features that increase the prediction probability for the target class) to a Red channel. In contrast, negative values (indicating features that suppress or decrease the prediction) were mapped to a Blue channel. Finally, we applied an alpha-blending technique, where the opacity of the color overlay was proportional to the absolute magnitude of the feature’s importance (capped at 70% maximum opacity). This ensures the underlying cellular morphology remains visible while clearly distinguishing regions that positively drive the classification from those that negatively influence it.

H. Implementation Environment

All experiments were implemented in MATLAB R2024b (The MathWorks, Natick, MA, USA) with the Deep Learning Toolbox. Training was performed on an NVIDIA [GTX 1650] GPU with CUDA version [12.1]. We set a fixed random seed (rng(42)) before all data partitioning and network initialization to ensure full reproducibility. The complete source code is provided as Supplementary Material.

## 3. Results

We evaluated the proposed CNN model’s performance in classifying (ALL) subtypes from peripheral blood smear images. We evaluated the model using hold-out testing and 5-fold cross-validation. We present the results using confusion matrices, overall classification accuracy, Receiver Operating Characteristic (ROC) curves, upsampled feature map activations, occlusion sensitivity, and SHAP-style explanation overlay analysis. We used explainable AI techniques to highlight the image regions that contributed most to the CNN predictions and improve the interpretability of the classification process. We implemented all experiments in MATLAB using the Deep Learning Toolbox in a GPU-accelerated environment. The study was conducted on both raw (Non-segmented) and Segmented image datasets to further evaluate the effect of cell segmentation on classification performance.

A. Computational Complexity and Efficiency

The proposed CNN contains 58,028 trainable parameters and requires approximately 211.44 Million FLOPs per inference. The model occupies 0.43 MB on disk. The average GPU inference time was 0.004 ± 0.0003 s per image. Eliminating the HSV segmentation preprocessing step further reduces the total pipeline computational cost by removing the thresholding, morphological operations, and masking stages.

B. Hold-out Validation Results

**Figure 3:**
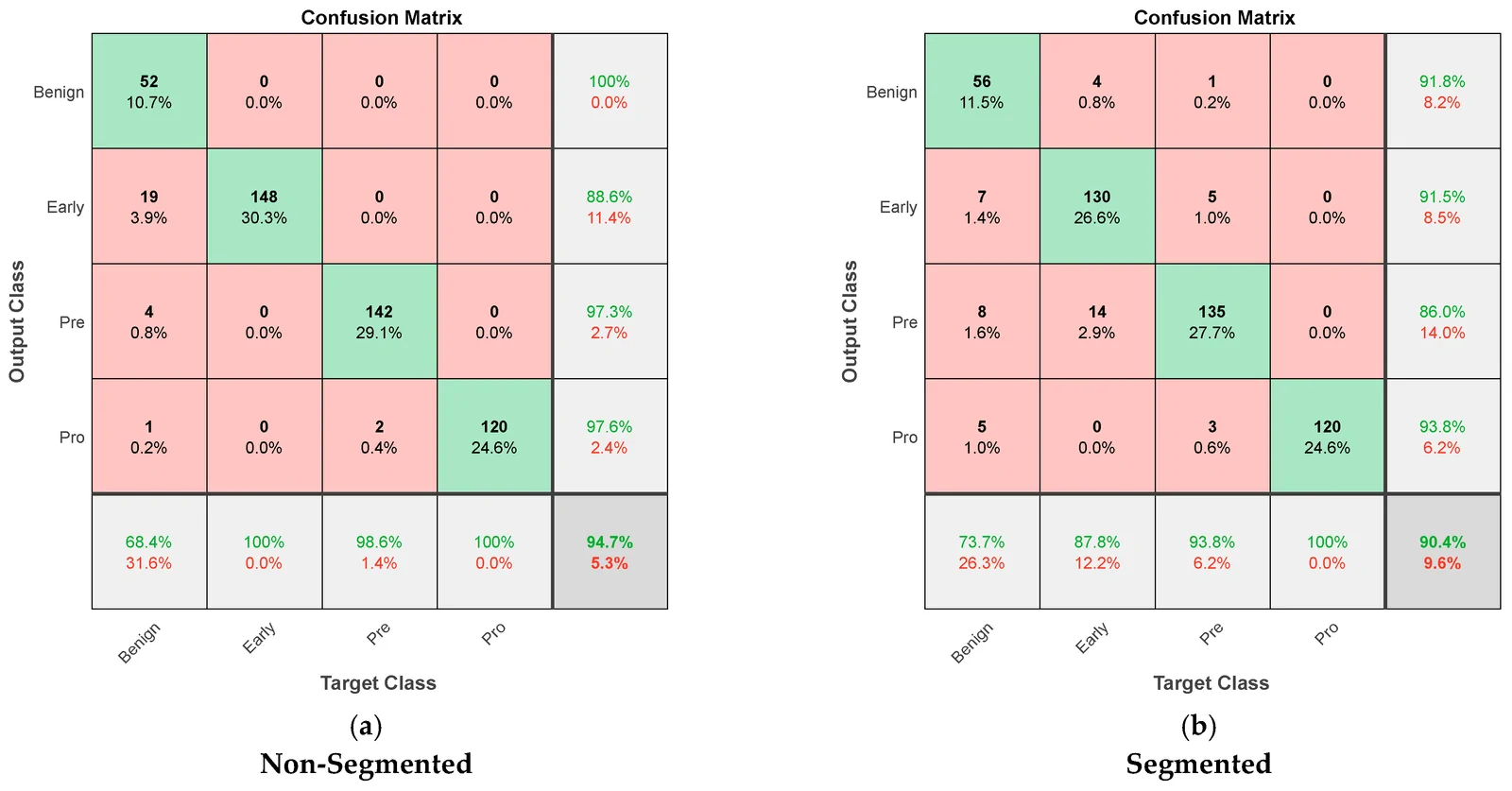
Confusion matrix of the proposed CNN model for classification of (**a**) Non-segmented PBS images, (**b**) Segmented PBS images into Benign, Early, Pre, and Pro classes.

The confusion matrices in Figure 3 show the proposed CNN model’s classification performance on the strictly held-out test set for both Non-segmented and Segmented PBS images. For the Non-segmented images, the model achieved a robust overall accuracy of 94.7%, with macro-averaged sensitivity, specificity, precision, and F1-scores of 91.76%, 98.11%, 95.86%, and 92.98%, respectively. These results indicate that the proposed CNN successfully learned important morphological features directly from the original images, even in the presence of background information. In contrast, when evaluated on the Segmented images, the model’s performance declined notably, achieving an overall accuracy of 90.4%, with macro-averaged sensitivity, specificity, precision, and F1-scores of 88.82%, 96.67%, 90.77%, and 89.47%, respectively. The main diagonal of both confusion matrices contains a high concentration of correctly classified samples, confirming the model’s strong predictive capability. However, the drop in performance for the Segmented variant, particularly in sensitivity and F1-score, suggests that removing surrounding cellular and contextual information during HSV segmentation may have discarded diagnostically relevant features. Most classification errors in both variants occurred between the Benign and Early subtypes, likely because certain early-stage leukemia blasts morphologically resemble normal hematogones in blood smear images.

**Figure 4:**
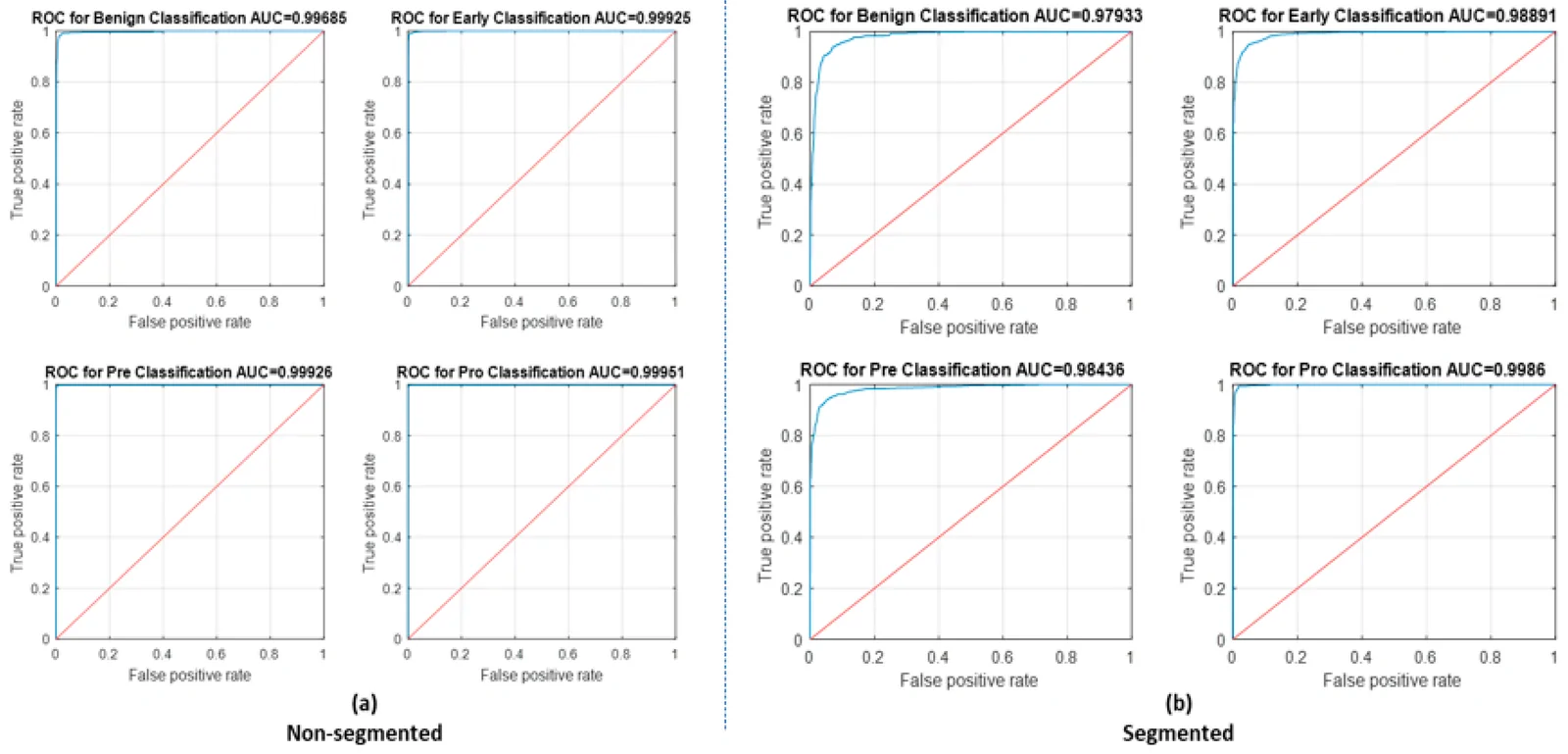
ROC-based performance evaluation across four leukemia subtypes.

Per-class ROC analysis was also performed to assess the proposed CNN model to differentiate among ALL subtypes, using one-vs-rest approach. As illustrated in Figure 4a, a near-ideal classifier behavior was achieved in raw, Non-segmented images, with ROC curves approaching the upper-left corner, indicating high sensitivity and specificity across all subtypes. Moreover, the corresponding AUC values were close to 1.0, reflecting a strong discriminative capability of the proposed CNN model. Pre and pro subtypes displayed the best ROC performance, suggesting that the model is able to distinguish easily between these two classes. Despite the Benign and Early classes showing strong performance, slight deviations from the optimal curve were noticed, which suggests a minor overlap in their learned feature representations and is consistent with the misclassifications observed in the confusion matrix. The analysis was also performed using the same one-vs-rest analysis as on Non-segmented images. Figure 4b illustrates the ROC curves for each subtype. Curves in this dataset were also positioned above the diagonal reference line, indicating good classification performance across all four classes despite the reduction in overall accuracy relative to the Non-segmented dataset. It is also evident that the Pro subtype showed the best classification performance, then the Early subtype, and followed by the Pre subtype. The Benign class showed the lowest performance, which is consistent with the higher level of misclassification observed in the confusion matrix.

C. Stratified 5-Fold Cross-Validation Results

Metrics across 5-stratified folds are represented in Table 5. Non-segmented images performed better than segmented images across all measures. For this segmentation preprocessing step, HSV-based segmentation did not improve classification performance. Non-segmented images provided stable classification performance, suggesting that using the original image information was better for distinguishing the four ALL subtypes under the evaluated CNN framework.

We conducted a paired t-test on the fold-wise metrics to assess statistical significance. The Non-segmented pipeline showed statistically significant superiority over the Segmented pipeline for both Accuracy (*p* = 0.0001) and F1-Score (*p* = 0.0001), confirming that the observed performance difference is consistent across data partitions.

Per-class metrics shown in Table 6 reflect the proposed CNN’s classification performance for the four classes under the Non-Segmentation and Segmentation settings. The results show that the Non-segmented representation performed better across all four classes. Pro-B ALL performed best, while Early and Pre-B showed the largest metric decreases after segmentation. The AUC values for these classes suggest that HSV segmentation removed or altered image characteristics that help distinguish morphologically similar subtypes. Moreover, the Benign subtype had high specificity but lower sensitivity and precision, indicating sample misclassifications.

D. Visualization Analysis: Upsampled feature map activation, occlusion sensitivity, and SHAP-style explanation overlay

**Figure 5:**
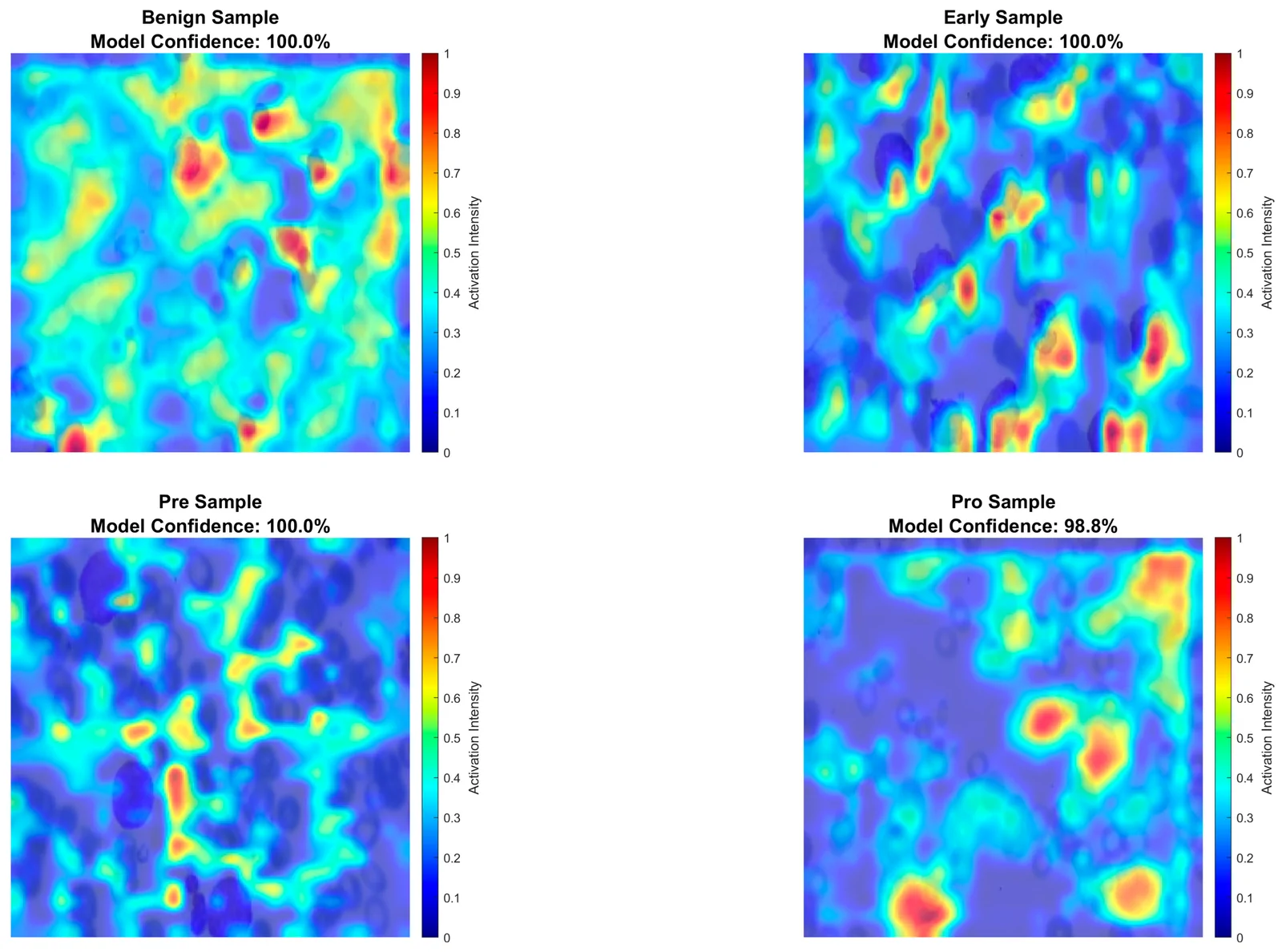
Upsampled feature map activations (upsampled to 224 × 224) of representative Non-segmented PBS Images for four ALL subtypes. each annotated with the corresponding Softmax probability. The model correctly predicts each subtype with 100% confidence; upsampled feature map activations were derived from the final convolution layer, min–max-normalized, and threshold; warmer colors denote higher activation.

To understand the proposed model’s decision-making process, we generated upsampled feature map activations (to 224 × 224) from the final convolutional layer to visualize the regions that contribute to the CNN’s predictions. After normalization and thresholding, the upsampled feature map activations focused only on regions that contribute to the most discriminative features used by the network. In Non-segmented images, the upsampled activation maps revealed that leukocyte-related morphological structures, such as nuclear regions, boundaries, and staining characteristics were the region with the primary focus rather than irrelevant background areas. Figure 5 shows four examples of the model’s upsampled feature map activation visualizations for all subtypes. The model correctly predicts each example as its respective subtype: “Benign,” “Early,” “Pre-B,” and “Pro-B.” The upsampled feature map activations highlight the regions of the cells that contribute most strongly to the model’s predictions, visually representing the features used to distinguish among the four subtypes.

**Figure 6:**
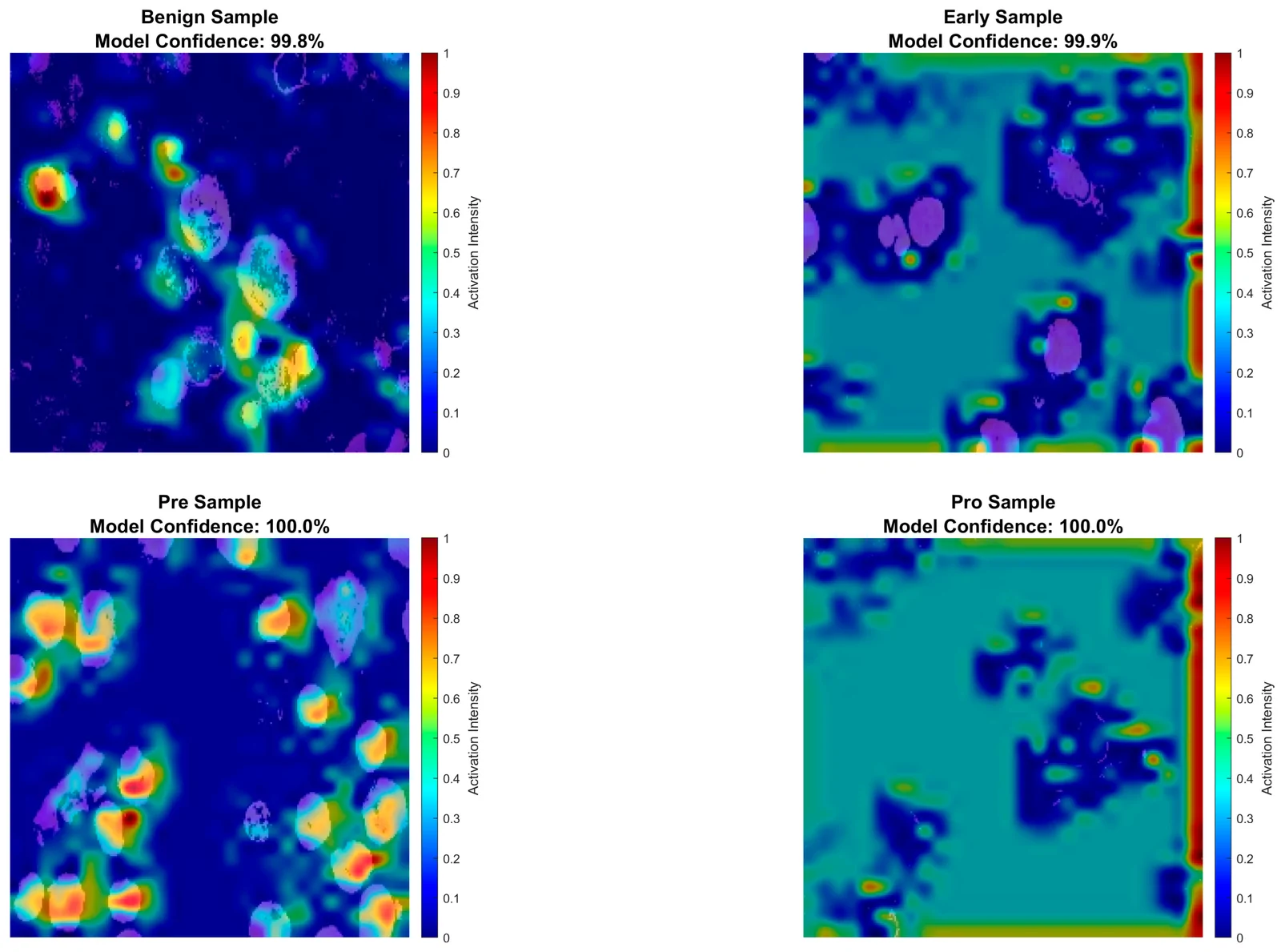
Upsampled feature map activations (upsampled to 224 × 224) for 4 ALL subtypes after HSV color threshold segmentation. Activation is confined to the isolated leukocyte regions. The model again predicts the subtypes correctly with reduced confidence for the Benign and Early subtypes.

In contrast, when generated using the segmented image dataset, the model focused more on isolated cell information than on contextual information because the background was removed. Figure 6 shows examples of the model’s upsampled feature map activations for four ALL subtypes, with the addition of a segmentation step. As shown, the model also predicts them correctly but shows lower confidence scores for the Benign and Early subtypes.

**Figure 7:**
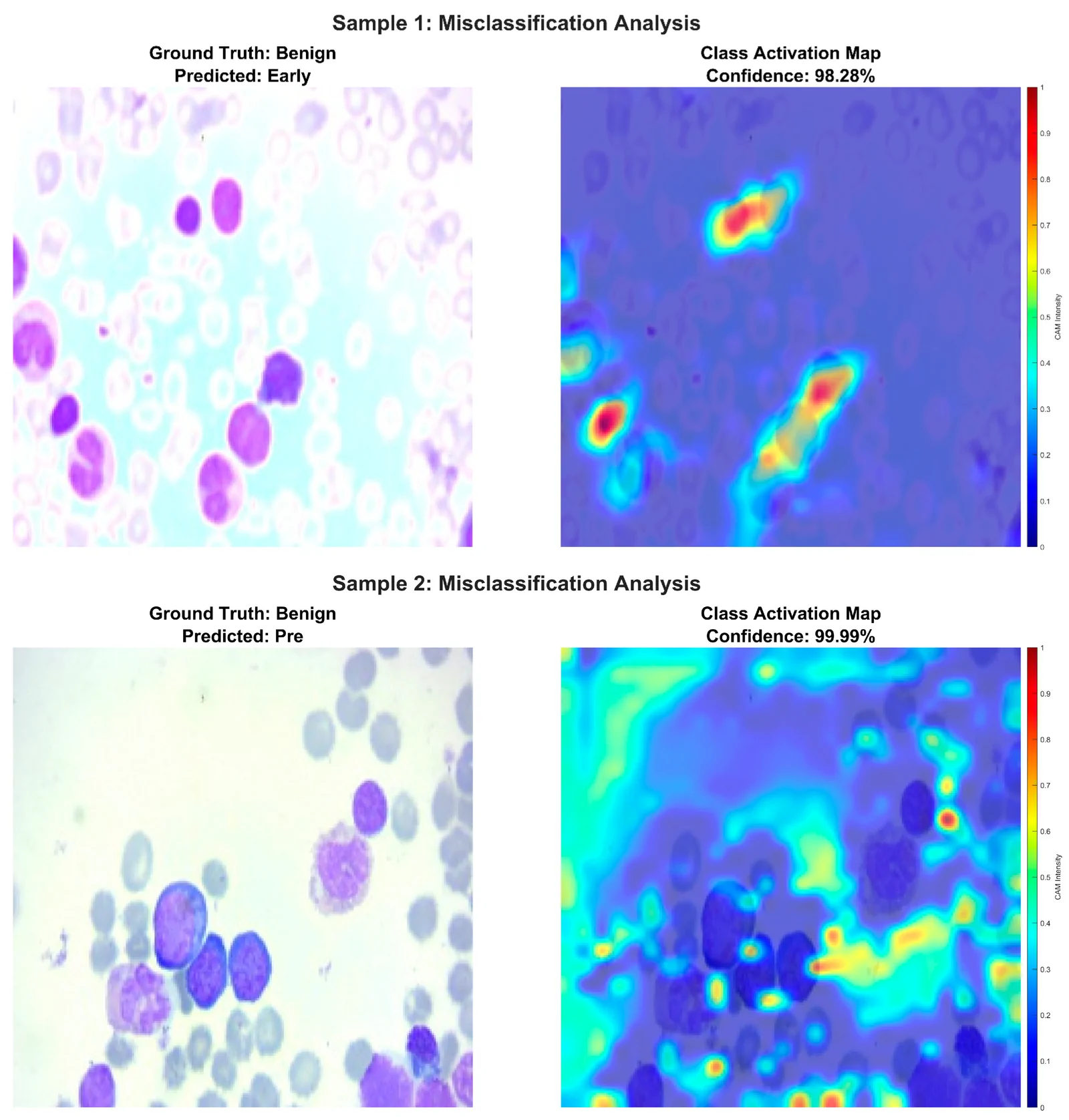
Upsampled feature map activations (upsampled to 224 × 224) for two misclassified Non-segmented (PBS) images. Left, original image; Right, corresponding upsampled feature map activation overlay. Rows show true Benign predicted as Early Pre-B ALL (98.28%) and true Benign predicted as Pre-B ALL (99.99%); percentages are the confidence assigned to the incorrectly predicted class.

**Figure 8:**
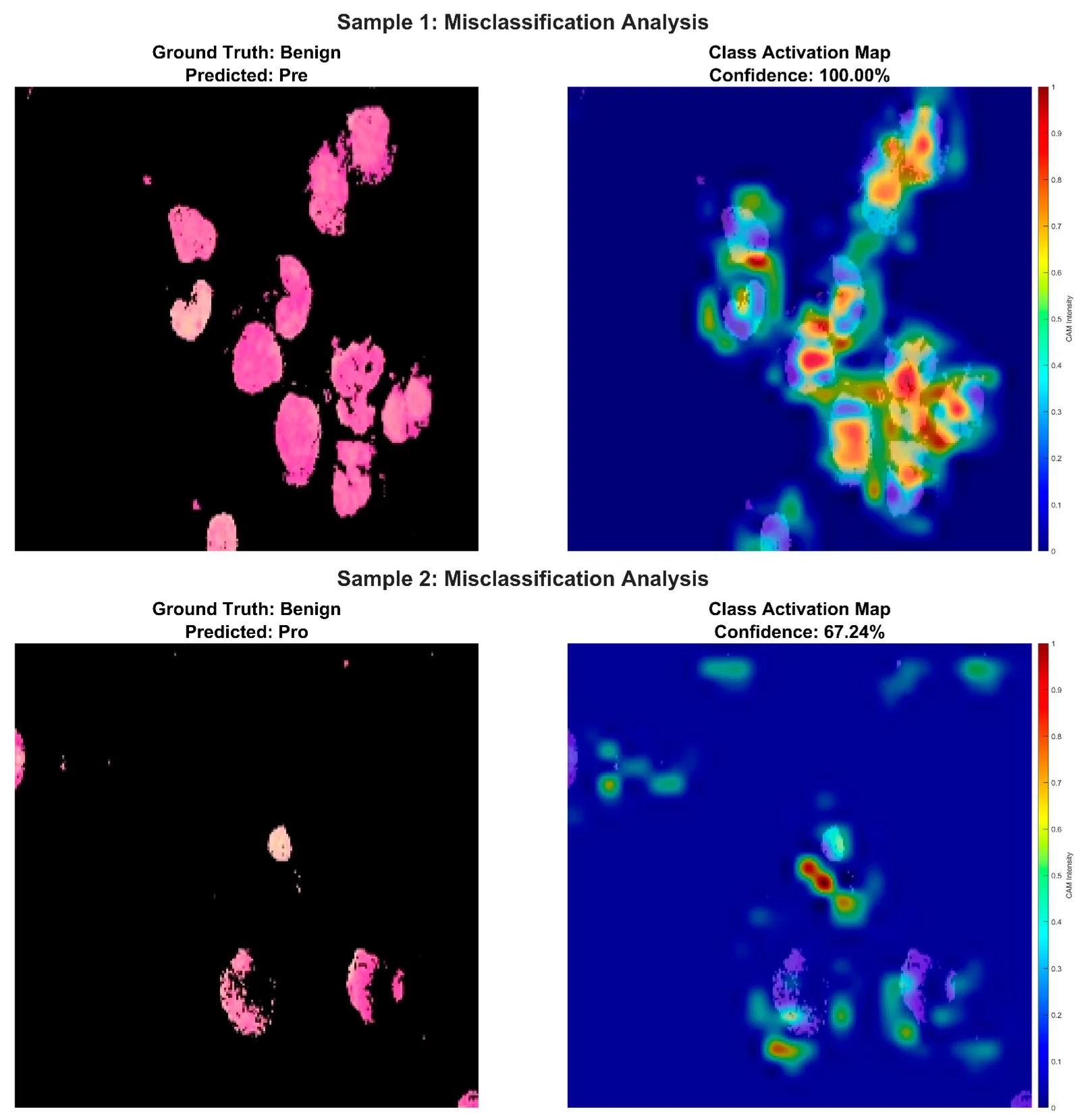
Upsampled feature map activations (upsampled to 224 × 224) of the Segmented dataset. Left, original image; Right, corresponding upsampled feature map activation. Rows show true Benign predicted as Pre-B ALL (100%) and true Benign predicted as Pro-B ALL (67.24%); percentages are the confidence assigned to the incorrectly predicted class.

To further understand the model’s behavior in cases where misclassifications occurred, Figure 7 demonstrates upsampled feature map activation visualizations for some misclassified Non-segmented PBS images. As shown, the Benign class was predicted as Early or Pre, which aligns with the confusion matrix results showing that 19 benign images were predicted as Early. In comparison, 4 images were assigned to the PRE-B All class (Figure 3a). The same applies to Segmented images (Figure 8), where the proposed model focuses on Segmented leukocyte regions that contain discriminative features rather than irrelevant areas. Closer inspection shows that these misclassifications align with the confusion matrix results in Figure 3b, where Benign Segmented images were classified as pre-B and pro-B ALL. Several misclassifications also occurred with high confidence, up to 100% in the Segmented dataset.

**Figure 9:**
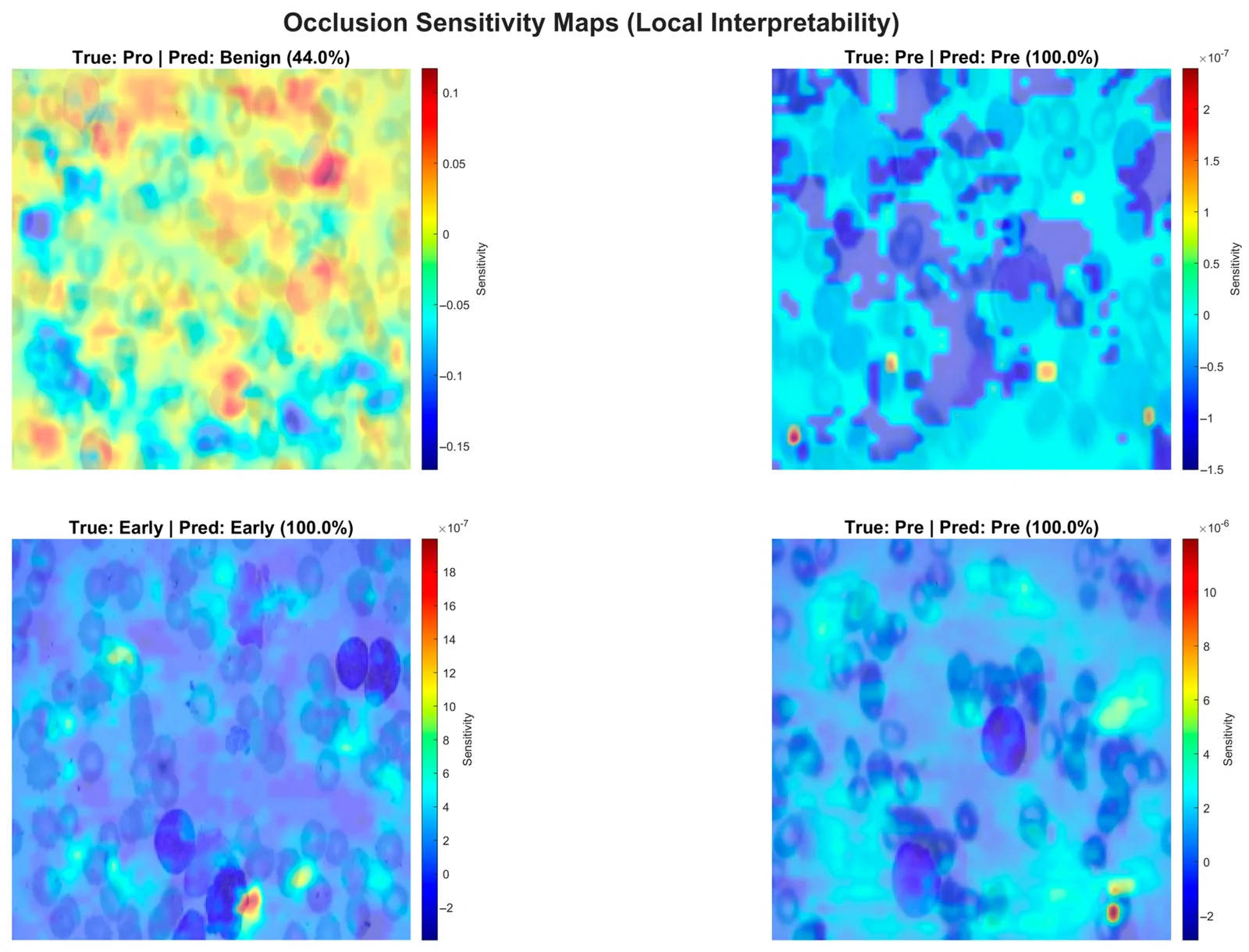
Occlusion sensitivity maps for representative test Non-segmented samples. Red regions indicate areas where masking caused the largest decrease in predicted class confidence.

To evaluate local feature importance through perturbation, we used occlusion sensitivity analysis on both Non-segmented and Segmented images. Figure 9 shows the occlusion sensitivity maps for representative test samples of Non-segmented. The model correctly classified the Early and Pre samples with 100% confidence. In contrast, the model incorrectly classified the Pro sample as Benign with a confidence of 44.0%, indicating a low-confidence classification.

The Early and Pre samples exhibited the clearest explanation. The model achieved 100% confidence, with low to negative sensitivity across the image and several localized regions of higher sensitivity. These regions correspond to areas that had a greater influence on the model’s prediction, indicating that the network has learned highly discriminative representations for the Early stage.

Unlike the previous classes, the Pro sample is misclassified as Benign with a low confidence score of 44.0%. The occlusion sensitivity map shows greater areas with high sensitivity. This behavior suggests that the classifier based its decision on limited visual evidence that resembles benign morphological patterns. The incorrect prediction may result from overlapping cellular characteristics between the Pro and Benign classes, intra-class variability, or insufficient discriminative features within this particular image.

**Figure 10:**
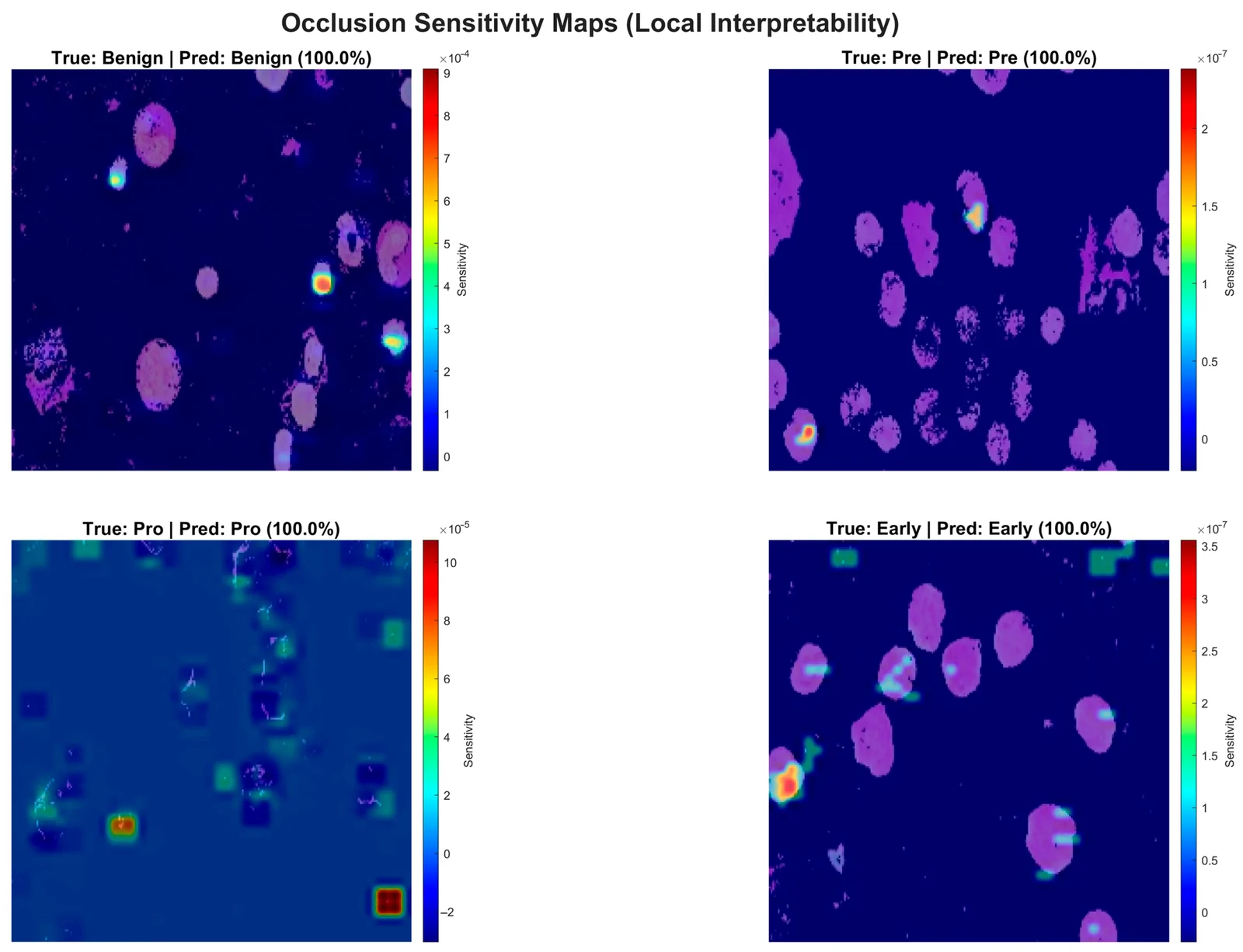
Occlusion sensitivity maps for representative test Segmented samples. Red regions indicate areas where masking caused the largest decrease in predicted class confidence.

For the Segmented samples shown in Figure 10, the model correctly classified the 4 subtypes with confidence scores of 100%. Applying HSV segmentation eliminated background information, allowing the model to focus on relevant cell features rather than other contextual information.

Overall, the occlusion sensitivity visualizations show that the proposed model generally focuses on biologically meaningful cellular structures rather than irrelevant background information. However, the misclassification of the Pro sample shows that certain advanced-stage images remain challenging, particularly when their morphology overlaps with benign cases. These findings emphasize the importance of incorporating explainability techniques alongside quantitative performance metrics, as they provide valuable insight into the model’s reasoning process and help identify potential weaknesses that can guide future improvements.

To further validate that the proposed CNN relies on biologically relevant morphological features rather than background artifacts or staining inconsistencies, we employed a SHAP-style explanation overlay visualization technique. Unlike standard Class Activation Maps (CAMs), which typically highlight broad regions of interest, this approach uses a diverging color scale (Red/Blue) to distinguish features that positively contribute to the predicted class (Red) from those that negatively contribute or suppress the prediction (Blue).

**Figure 11:**
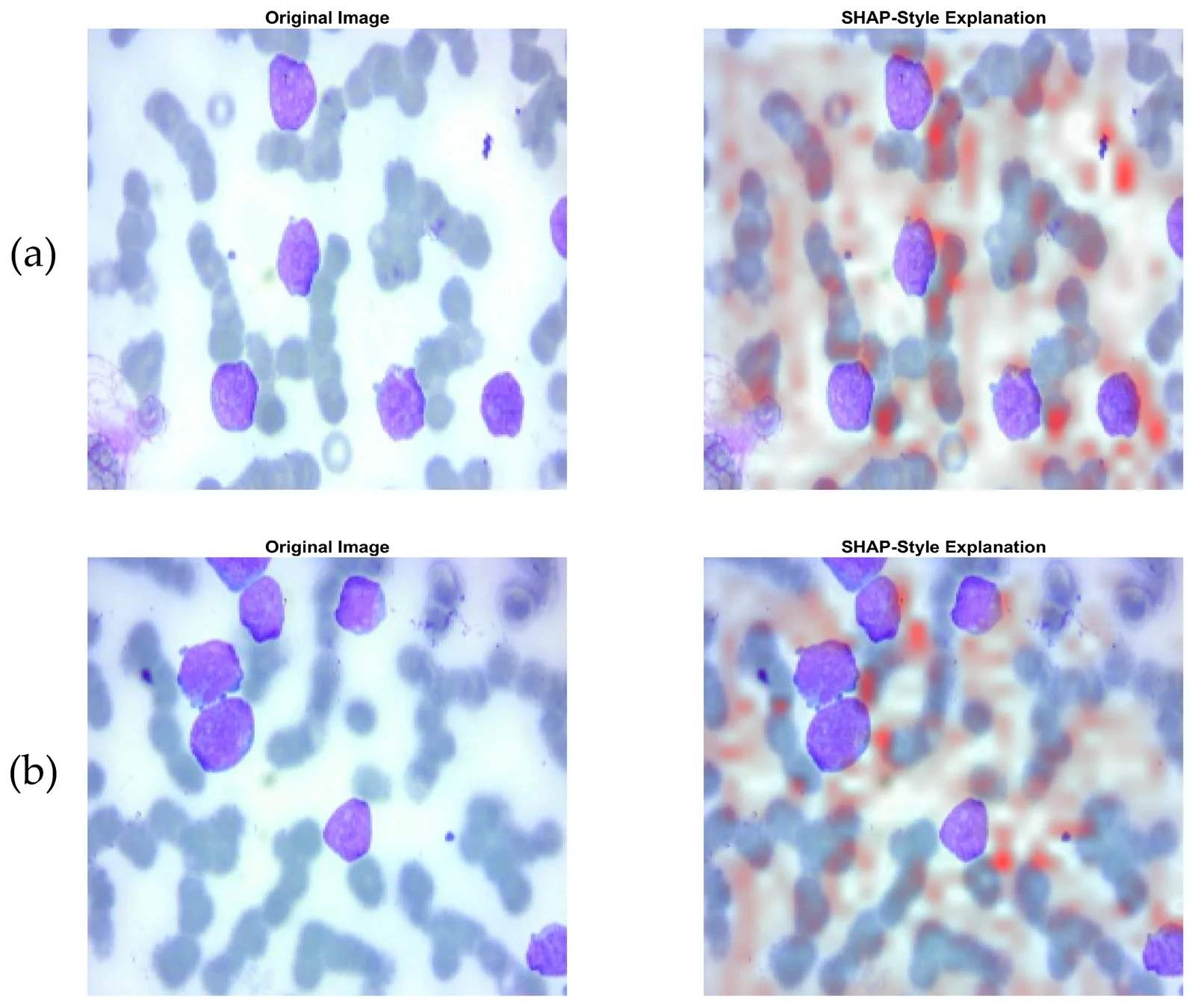
An example of SHAP-style explanation overlay visualization for an early pre-B ALL cell. (**a**) Distributed positive attribution (red) highlights nuclear regions and surrounding erythrocyte clusters; blue regions indicate suppression of irrelevant background. (**b**) Localized activation (deep red) concentrated on nuclear boundaries and chromatin texture of the central blast cell.

Figure 11 illustrates the explainability analysis for a representative sample from the Early Pre-B ALL class. In the first visualization (Figure 11a), the explanation map reveals a distributed pattern of positive attribution (red regions) across the field of view. Notably, the model assigns high positive importance not only to the distinct, large purple nuclei characteristic of lymphoblasts but also to specific clusters of surrounding erythrocytes (red blood cells). This suggests that for the “Early” subtype, the network leverages contextual information, specifically the spatial relationship and density of cells within the smear, to inform its decision, rather than isolating the blast cell in a vacuum. The blue regions in the background indicate that the model actively suppresses irrelevant white space or non-cellular artifacts, confirming it has learned to ignore noise. In the second visualization (Figure 11b), which represents a refined or alternative explanation view, the attribution becomes highly localized. Here, the positive activation (deep red) is intensely concentrated on the nuclear boundaries and chromatin texture of the central blast cells. This sharp focus confirms that the network has successfully learned the discriminative nuclear-to-cytoplasmic ratio and chromatin condensation patterns that are hallmarks of the Early Pre-B ALL diagnosis. The suppression of the surrounding background (indicated by the lack of red overlay in intercellular spaces) further demonstrates the model’s robustness against variations in slide preparation.

Collectively, these visualizations provide critical evidence that legitimate hematological features drive our lightweight CNN’s high accuracy (98.4%). The model does not merely detect “purple blobs”; it attends to specific morphological structures, nuclear SHAP-style explanation overlays, size, and cellular context that a human pathologist would use to distinguish ALL subtypes. This alignment between machine attention and clinical diagnostic criteria significantly enhances the trustworthiness of the proposed framework for potential clinical decision support.

E. Subject-Level Grouping Analysis and Proxy-Patient Cross-Validation

**Figure 12:**
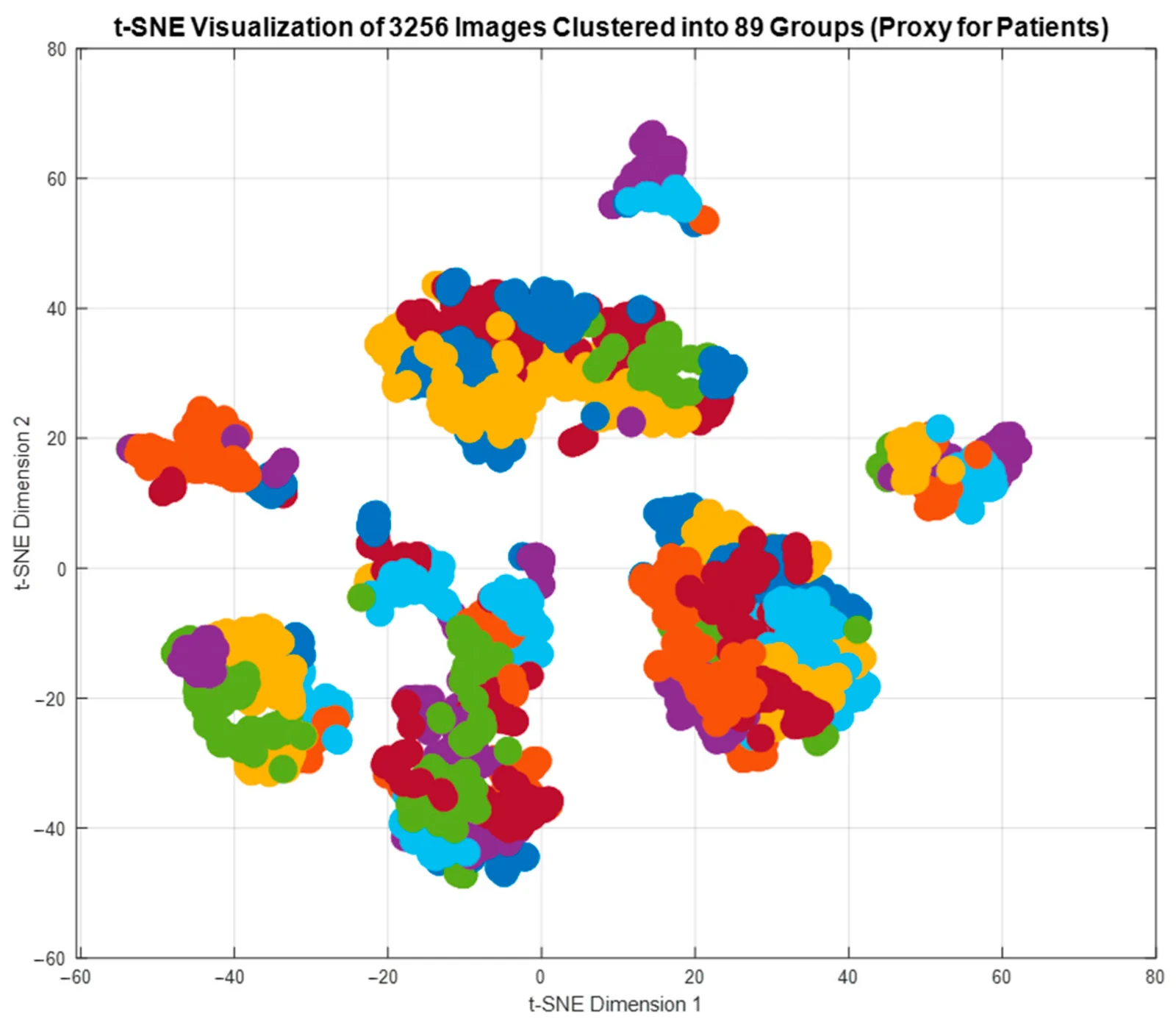
t-SNE visualization of the 3256 images clustered into 89 groups (proxy patients) based on 3D HSV color histograms. Distinct colored blobs represent unique slide/staining batches. Each color represents a patient.

To evaluate the feasibility of patient-disjoint splitting, we analyzed the composition of the 89 K-means clusters. The t-distributed Stochastic Neighbor Embedding (t-SNE) visualization (Figure 12) reveals that while the clustering algorithm successfully grouped images with highly similar staining and background characteristics, it did not perfectly isolate individual patients. Specifically, 20 out of the 89 clusters (22.5%) contained a mixture of both Benign (healthy) and Malignant (ALL) images (Table 7). This mixing clearly shows that the unsupervised algorithm groups images by slide preparation and staining batch rather than strict patient identity.

Despite this limitation, the Subject-Level Grouped 5-Fold CV provides a rigorous, leakage-minimized evaluation (Table 8). Under these strict constraints, the Non-segmented pipeline still demonstrated statistically significant superiority over the Segmented pipeline. The Non-segmented images achieved a mean subject-wise accuracy of 97.87% ± 0.54%, whereas the Segmented images achieved a mean accuracy of 89.66% ± 4.01% (Table 9). Although the overall accuracy values were slightly lower than those observed in the image-level stratified CV (98.77% vs. 91.34%), as expected given the increased difficulty of grouped evaluation, the fundamental trend remained identical. This confirms that the superiority of the Non-segmented pipeline is a robust biological finding, not an artifact of image-level data leakage.

## 4. Discussion

This study evaluated a custom CNN to classify four categories in a public B-ALL image dataset: Benign (hematogone), Early Pre-B ALL, Pre-B ALL, and Pro-B ALL. The principal analysis compared the original PBS images with their paired HSV-segmented versions supplied by the dataset creators, while keeping the classification architecture and experimental conditions unchanged.

Under the evaluation protocol, accuracy was 98.8% with the Non-segmented images and 91.3% with the supplied Segmented images. Sensitivity also decreased across the four categories when the supplied Segmented images were used. The greatest reduction occurred for Benign images, with an increased confusion between Early Pre-B ALL and Pre-B ALL. A possible explanation is that the HSV-segmented representation removed contextual or staining information used by this CNN. Poor segmentation quality and imperfect masks were reported to eliminate surrounding valuable features that contribute to the learning process and, in this case, affect feature extraction and subtype recognition [55]. Hence, the model performed better when trained and evaluated on the Non-segmented peripheral blood smear images.

The Pro-B ALL subtype was also the most accurately classified, suggesting that morphological characteristics at this stage were more distinct and easier for the CNN to recognize. In contrast, the largest decrease was seen in the Benign class, which appears to have a wider range of morphological and contextual features, making it more vulnerable to information loss during segmentation. This interpretation remains a hypothesis because the study did not compare alternative masks or segmentation algorithms. Accordingly, the result shows a difference between the two image representations; it does not show that segmentation is generally detrimental or unnecessary.

The confusion matrices provided further insight into the different error patterns. Errors with the original images were concentrated mainly in the Benign category, whereas the HSV-segmented images produced greater confusion between Early Pre-B ALL and Pre-B ALL. These categories represent related stages of B-lymphoblast development and can share morphological characteristics [50,56,57,58]. Removing surrounding context may therefore have affected cues used by the classifier, suggesting that the loss of contextual information, rather than segmentation itself, may have contributed to the increased Early–Pre confusion, which is consistent with work showing that combining Segmented regions with contextual background can improve classification relative to segmentation alone [59]. Nevertheless, this explanation is inferential. A direct comparison with alternative segmentation methods would be required to determine whether the observed change resulted from HSV segmentation specifically, mask quality, or loss of contextual information.

Previous studies reporting high classification performance used different CNN architectures, feature-selection methods, and hybrid approaches, as well as different dataset compositions, class definitions, and image acquisition protocols; thus, direct benchmarking against studies such as Alkhouli & Joshi [27] (2024, 99.56% on ALL-IDB) or Diaz Resendiz et al. [36] (2023, 99.9% on ALL-IDB) is not feasible. For example, the dataset creators previously evaluated the same image collection using a two-channel framework that combined original and HSV-segmented inputs and compared ten established CNN architectures, with DenseNet201 providing their best result [50]. In comparison, the present study applies one custom CNN separately to the original and HSV-segmented collections so we can examine the effect of the image representation while holding the classifier constant. Because the model architectures and validation procedures differ, the reported accuracy values cannot be interpreted as a direct performance benchmark.

We generated upsampled feature map activations, occlusion sensitivity maps, and SHAP-style explanation overlays to provide a qualitative view of the regions affecting model predictions. The visualizations suggest that the CNN frequently focused on relevant cellular structures, such as nuclear regions, boundaries, and staining characteristics, rather than irrelevant background artifacts. Still, upsampled feature map activations, occlusion sensitivity, and SHAP-style explanation overlays offered qualitative insight into visualizations and selected predictions and errors. Some highlighted regions overlapped visually salient cells, whereas other examples showed weak or dispersed activation, and several errors received high Softmax scores. These visualizations cannot, by themselves, establish that the network relied on biologically diagnostic morphology; they describe selected model behaviors and generate hypotheses, not demonstrate clinical reliability or readiness for deployment.

High-confidence misclassifications (e.g., a Benign sample incorrectly classified as Pre with 99.99% confidence) carry significant clinical implications. In a clinical decision-support setting, a highly confident but incorrect prediction is more dangerous than an uncertain one, as it may lead to false reassurance and delayed treatment. This highlights the risk of model overconfidence when confronted with morphologically ambiguous samples. While the model achieves excellent overall accuracy, its raw Softmax outputs should not be interpreted as calibrated probabilities without post hoc calibration techniques such as temperature scaling. Future work must evaluate prediction calibration and confidence reliability before clinical implementation.

Nevertheless, interpret the findings within the boundaries of this design. First, all images came from a single public dataset. Although its paired structure enabled comparison of the Non-segmented and HSV-segmented representations using the same classification architecture and experimental conditions, the model’s generalizability to images acquired under different staining protocols, microscopy systems, and laboratory conditions remains unverified. External validation on independent, multi-center datasets is essential before clinical applicability can be established [60,61]. Also, the segmentation analysis was limited to HSV color-thresholding provided by the dataset creators, and no quantitative segmentation quality metrics (e.g., Dice coefficient, Intersection-over-Union) could be computed because ground-truth segmentation masks were unavailable. Future studies should evaluate alternative segmentation methods, particularly deep learning-based approaches, with quantitative quality benchmarks.

Second, a critical methodological limitation is the risk of patient-level data leakage. The dataset comprises 3256 images from 89 patients; however, the public release does not provide patient-level identifiers linking images to individual patients. To mitigate this risk and evaluate the model’s true generalizability, we implemented subject-level (Grouped) 5-fold cross-validation. Since explicit patient metadata was unavailable, we utilized unsupervised K-means clustering on HSV color histograms to group the 3256 images into 89 distinct clusters, serving as “proxy patients” based on shared slide preparation and staining characteristics. We enforced a strict grouped split ensuring that all images from a single proxy-patient cluster were confined exclusively to either the training or testing sets, thereby eliminating image-level leakage. Under these strict, leakage-minimized conditions, the Non-segmented pipeline maintained a statistically significant performance advantage, achieving a mean subject-wise accuracy of 97.87% ± 0.54%, compared to 89.66% ± 4.01% for the Segmented pipeline (*p* < 0.001). Although overall accuracy was slightly lower than in the standard image-level stratified CV—as expected, given the increased difficulty of grouped evaluation—the fundamental trend remained the same. This finding confirms that the Non-segmented pipeline’s superiority is a robust feature-level phenomenon, not an artifact of patient-level data leakage. It further reinforces the conclusion that the HSV-based segmentation process discards vital contextual morphological cues required to distinguish closely related ALL subtypes, even when the model is forced to generalize across entirely unseen proxy-patient groups.

Third, this study did not quantitatively validate explainability visualizations with an experienced hematologist or pathologist. Future studies should incorporate expert assessment to confirm that the model’s attention regions align with clinically established diagnostic criteria. Finally, several incorrect predictions received high Softmax scores, showing that confidence alone did not reliably identify errors. Finally, the post hoc visualizations provide qualitative insight into selected examples, but their spatial resolution and example-based nature do not support precise biological localization or clinical validation. These considerations define the next steps for evaluating the model under broader and more clinically representative conditions.

Taken together, the paired comparison indicates that this CNN performed better with the original PBS images than with the HSV-segmented images supplied in the public dataset. The result suggests that the supplied Segmented representation did not benefit this classifier and may have removed useful contextual information. The finding is specific to the evaluated dataset and segmentation output and should not be generalized to other segmentation methods. Overall, the presented work supports the growing potential of deep learning techniques for automated leukemia detection and subtype classification from peripheral blood smear images.

Finally, to address the clinical safety implications of high-confidence misclassifications, we evaluated model calibration using expected calibration error (ECE). The Non-segmented pipeline achieved an ECE of 0.0455, whereas the Segmented pipeline yielded a higher ECE of 0.0525. This indicates that the HSV segmentation process not only reduces overall accuracy but also degrades the reliability of the model’s probability estimates. In a clinical decision-support setting, do not interpret raw Softmax outputs as calibrated probabilities without post hoc techniques such as temperature scaling, which must be evaluated in future deployments.

## 5. Conclusions

The study presents a lightweight CNN model for the automated classification of ALL subtypes from PBS images. The model was designed to classify four categories: Benign, Early Pre-B ALL, Pre-B ALL, and Pro-B ALL. We evaluated performance using hold-out validation, 5-fold cross-validation, and rigorous Subject-Level Grouped Cross-Validation to mitigate patient-level data leakage. We assessed explainability using upsampled feature map activations, occlusion sensitivity, and SHAP-style explanation overlay visualizations. The proposed model achieved 98.8% accuracy using Non-segmented images, while HSV-segmented images achieved 91.3% accuracy. Crucially, under strict Subject-Level Grouped 5-Fold Cross-Validation, the Non-segmented pipeline remained statistically superior (97.87% vs. 89.66%), confirming that the performance degradation caused by HSV segmentation is a robust feature-level phenomenon rather than an artifact of image-level data leakage. The comparative analysis shows that the HSV-based segmentation evaluated in this study degrades classification performance because it loses contextual morphological features. This finding does not establish that segmentation is universally unnecessary; rather, it indicates that the thresholding method used here discards diagnostically relevant information. Overall, the proposed model supports the growing potential of using deep learning techniques as supportive tools for computer-based leukemia diagnosis. Future studies should investigate advanced boundary-aware segmentation (e.g., U-Net) to determine if high-fidelity masks can outperform the Non-segmented baseline. Furthermore, because the public dataset lacks explicit patient-to-image metadata, future work must prioritize external validation on truly patient-disjoint cohorts to establish definitive clinical generalizability.

## Figures and Tables

**Table 1:** Representative traditional ML, hybrid, and CNN-based leukemia detection studies.

Category	Study	Method	Reported Accuracy	Dataset
Traditional ML	Putzu et al. (2014) [30]	SVM (RBF) on hand-crafted shape/color/texture features	93% (98% sensitivity)	Custom (33 images)
Traditional ML	Rawat et al. (2015) [31]	GLCM texture + shape features → SVM	89.8%	Custom
Traditional ML	Saikia et al. (2024) [32]	CIELab K-means clustering + Whale-Optimized SVM	98.42%	ALL-IDB1
Hybrid (Deep features + ML classifier)	Kumar et al. (2025) [33]	CNN feature extraction + SVM classifier	95.8%	ALL-IDB1/IDB2
Hybrid (Deep features + ML classifier)	Sulaiman et al. (2023) [34]	ResNet50 features + Random Forest selection + SVM	90.0%	Blood smear (custom)
CNN-based	Alkhouli & Joshi (2024) [27]	Optimized lightweight CNN	99.56% (F1 99.53%)	ALL-IDB1/IDB2
CNN-based	Jawahar et al. (2022)—ALNett [35]	Cluster-layer deep CNN	91.13% (F1 0.96)	Microscopic WBC images
CNN-based	Diaz Resendiz et al. (2023) [36]	U-Net segmentation + ResNet-50	99.9%	Six public databases

**Table 2:** Class distribution of the ALL image dataset (Aria et al., 2021) [49].

Class	ALL Subtype	Image Count	Proportion (%)
Benign	Hematogone (non-cancerous)	504	15.48%
Early	Early Pre-B ALL	985	30.25%
Pre	Pre-B ALL	963	29.58%
Pro	Pro-B ALL	804	24.69%
Total	—	3256	100%

**Table 3:** CNN training hyperparameters. Summary of the optimizer, learning rate, batch size, and hold-out/5-fold cross-validation settings used to train the proposed CNN.

Hyperparameter	Value/Setting
Optimizer	Adam
Initial Learning Rate	0.001
Maximum Epochs	100
Mini-Batch Size	64
Data Shuffle	Every epoch
Validation Frequency	Every 50 iterations
Execution Environment	GPU (CUDA)
Hold-out Split (Train/Val/Test)	70%/15%/15%
K-Fold Cross-Validation	5-fold (systematic partition)
Input Image Size	224 × 224 × 3 (RGB)

**Table 4:** Performance evaluation metrics. Formulas for the classification metrics (accuracy, sensitivity, specificity, precision, F1-score, and AUC) used to evaluate model performance.

Metric	Formula
Accuracy	(TP + TN)/(TP + TN + FP + FN)
Sensitivity (Recall)	TP/(TP + FN)
Specificity	TN/(TN + FP)
Precision (PPV)	TP/(TP + FP)
F1-Score	2·Precision·Recall/(Precision + Recall)
AUC	Area under per-class ROC curve (one-vs-rest)

**Table 5:** Stratified 5-fold cross-validation performance (mean ± SD, 95% CI).

Metric	Non-Segmented (Mean ± SD)	95% CI	Segmented (Mean ± SD)	95% CI
Accuracy	98.77 ± 0.38%	[98.30–99.24%]	91.34 ± 0.87%	[90.26–92.42%]
Sensitivity	98.46 ± 0.63%	[97.67–99.25%]	90.38 ± 0.88%	[89.29–91.47%]
Specificity	99.60 ± 0.13%	[99.44–99.76%]	97.07 ± 0.29%	[96.71–97.42%]
Precision	98.54 ± 0.44%	[97.99–99.09%]	90.87 ± 1.15%	[89.44–92.30%]
F1-Score	98.49 ± 0.50%	[97.87–99.12%]	90.55 ± 0.96%	[89.36–91.74%]

**Table 6:** Per-class performance metrics (mean across 5 stratified folds).

Non-Segmented						Segmented				
Class	Sensitivity (%)	Specificity (%)	Precision (%)	F1-Score (%)	AUC	Sensitivity (%)	Specificity (%)	Precision (%)	F1-Score (%)	AUC
Benign	95.84 (±2.92)	99.38 (±0.35)	96.64 (±1.87)	96.21 (±1.49)	0.998 (±0.002)	80.76 (±4.22)	97.60 (±0.89)	86.22 (±4.67)	83.32 (±3.41)	0.981 (±0.007)
Early	98.78 (±0.45)	99.47 (±0.40)	98.79 (±0.91)	98.78 (±0.45)	1.000 (±0.000)	89.85 (±2.27)	97.05 (±0.71)	92.96 (±1.68)	91.37 (±1.86)	0.988 (±0.005)
Pre	99.48 (±0.37)	99.83 (±0.24)	99.59 (±0.57)	99.53 (±0.34)	1.000 (±0.000)	92.42 (±2.76)	95.38 (±0.83)	89.3 (±1.66)	90.86 (±1.66)	0.984 (±0.006)
Pro	99.75 (±0.56)	99.71 (±0.31)	99.14 (±0.92)	99.44 (±0.51)	0.999 (±0.003)	98.51 (±1.29)	98.25 (±0.85)	94.90 (±2.38)	96.65 (±1.29)	0.998 (±0.001)
Macro Average	98.46 (±1.80)	99.60 (±0.21)	98.54 (±1.31)	98.49 (±1.56)	0.999 (±0.001)	90.38 (±7.37)	97.07 (±1.23)	90.87 (±3.85)	90.55 (±5.49)	0.988 (±0.008)
Weighted Average	98.77 (±0.38)	99.62 (±0.14)	98.78 (±0.38)	98.77 (±0.38)	0.999 (±0.001)	91.34 (±0.87)	96.93 (±0.30)	91.33 (±0.86)	91.28 (±0.85)	0.988 (±0.003)

**Table 7:** Class purity of the 89 proxy-patient clusters.

Cluster Composition	Number of Clusters	Percentage
Pure Benign	13	14.6%
Pure Malignant (Early/Pre/Pro)	56	62.9%
Mixed Benign & Malignant	20	22.5%
TOTAL	89	100.0%

**Table 8:** Subject and class distribution across five folds.

Fold	Subjects	Images	Benign	Early	Pre	Pro
1	17	726	9	276	185	256
2	18	641	75	106	119	341
3	18	411	111	166	81	53
4	18	750	199	221	238	92
5	18	728	110	216	340	62
Total	89	3256	504	985	963	804

**Table 9:** Subject-level 5-fold cross-validation performance (mean ± SD, 95% CI).

Metric	Non-Segmented 5-Fold CV (Mean ± SD) [95% CI]	Segmented 5-Fold CV (Mean ± SD) [95% CI]	Non-Segmented Hold-Out (Single Split %)	Segmented Hold-Out (Single Split %)
Accuracy	97.87 ± 0.54 [97.20–98.54]	89.66 ± 4.01 [84.68–94.64]	95.94	84.18
Sensitivity	97.45 ± 0.57 [96.74–98.16]	89.09 ± 4.29 [83.76–94.42]	96.35	80.07
Specificity	99.29 ± 0.19 [99.05–99.53]	96.48 ± 1.38 [94.77–98.19]	98.67	94.28
Precision	97.25 ± 1.43 [95.48–99.02]	88.61 ± 2.88 [85.03–92.19]	94.56	79.77
F1-Score	97.27 ± 0.81 [96.26–98.28]	88.40 ± 3.95 [83.49–93.31]	95.33	78.45

## Data Availability

Data is available online on Kaggle (DOI: 10.34740/KAGGLE/DSV/2175623).

## Supplementary Materials

The following supporting information can be downloaded at: https://github.com/aliqudah/ALL-Subtype-CNN-Segmentation-Impact (accessed on 20 December 2024).

## Abbreviations

The following abbreviations are used in this manuscript:

AI	Artificial Intelligence
ALL	Acute Lymphoblastic Leukemia
AML	Acute Myelogenous Leukemia
AUC	Area Under Curve
CAM	Class Activation Maps
CLL	Chronic Lymphocytic Leukemia
CML	Chronic Myelogenous Leukemia
CNN	Convolutional Neural Network
DL	Deep Learning
FN	False Negative
FP	False Positive
ML	Machine Learning
PBS	Peripheral Blood Smear
ROC	Receiver Operating Characteristic
SHAP	SHapley Additive exPlanations
SVM	Support Vector Machines
TN	True Negative
TP	True Positive
t-SNE	t-distributed Stochastic Neighbor Embedding
WBC	White Blood Cells
